# Comparative Study of Elabela and Apelin on Apelin Receptor Activation Through β-Arrestin Recruitment

**DOI:** 10.1007/s12033-022-00529-6

**Published:** 2022-08-12

**Authors:** Hong Zhang, Juan Chen, Min Shi, Feng Xu, Xiangcheng Zhang, Da-Wei Gong

**Affiliations:** 1 Department of Endocrinology, Huai’an First People’s Hospital, Nanjing Medical University, Huai’an, 223300 People’s Republic of China; 2 Division of Endocrinology, Diabetes and Nutrition, Department of Medicine, University of Maryland School of Medicine, Baltimore, MD 21201 USA; 3 Department of ICU, Huai’an First People’s Hospital, Nanjing Medical University, Huai’an, 223300 People’s Republic of China

**Keywords:** Elebela/Toddler, Apelin, The apelin receptor (APJ), ARRBs, NanoBiT®

## Abstract

Apelin receptor (APJ) ligands elabela (ELA) and apelin have divergent distributions and function differently in vitro and in vivo. Whether differences exist in their capacity of recruitment of β-arrestins (ARRBs) to APJ remains unknown. The aim of the current study was to investigate the different effects of ELA and apelin on the interaction between APJ and ARRBs in live cells by NanoBiT®. NanoBiT® system is a new technology for studying protein–protein interaction in real-time in live cells, based on the emission of luminescence when two split components of NanoLuc luciferase, large Bit (LgBit) and small Bit (SmBit), complement each other to form an enzymatically active entity. We tagged the APJ and ARRBs with LgBit or SmBit and then evaluated their interactions in transiently transfected HEK293T cells, and determined the signal strength yielded as a result of the interaction. We also investigated the concentration-dependent response of the APJ-ARRB interaction in response to ELA and apelin. Finally, we assessed the effect of F13A, an APJ antagonist which is structurally very similar to apelin-13, on ELA- and apelin-mediated APJ-ARRB interactions. The NanoLuc® luciferase signal was highest in the pair of APJ-LgBit with SmBit-ARRB1 or SmBit-ARRB2. NanoLuc® luciferase signal increased in a concentration-dependent manner from 0.1 nM to 10 μM in response to ELA or apelin. Interestingly, ELA elicited weaker APJ-ARRB interaction signals than apelin. Pre-treatment with F13A potently reduced the APJ-ARRB interaction in response to both ELA and apelin. Our results demonstrated that both ELA and apelin promoted the interaction of APJ and ARRBs in a concentration-dependent manner, and ELA is less efficacious than apelin in inducing the recruitment of ARRBs to APJ, providing a biased functional aspect of ELA vs. apelin at the receptor signaling level. Additionally, ELA and apelin may share the same binding site(s) or pocket(s) at the APJ level.

## Introduction

The apelin receptor (APJ) is a relatively ubiquitously expressed G protein-coupled receptor, paired with two endogenous ligands, apelin and elabela (ELA)/Toddler [1]. Apelin is widely expressed in the body, such as in the heart, lung, endothelial cells, kidney and brain [2, 3]. Apelin plays an important role in reducing blood pressure, enhancing myocardial contractility, promoting angiogenesis, regulating electrolyte balance, and promoting pituitary hormone release [2, 4–6]. In contrast. ELA is expressed only in a small number of tissues and organs, such as the kidney and heart [7, 8]. During the embryonic development, ELA plays a vital role in the development of the heart [9]. ELA deficiency leads to preeclampsia and cardiovascular malformations in mice [10]. Recently, ELA has been reported to be essential for heart development and stem cell maintenance and may play a protective role against renal injury [11, 12].

GPCRs primarily couple to four major Gα families, Gs, Gi/o, Gq/11, and G12/13, which dictate differential signaling cascades [13]. One of the most extensively studied G protein-independent signal transduction pathways is the coupling to the arrestin adaptor protein family. Of the four arrestin isoforms (arrestin 1–4), arrestin 1 and 4 are expressed in the visual system, whereas arrestin 2 (ARRB1) and arrestin 3 (ARRB2) are ubiquitously expressed. After short- or long-term agonist stimulation, β-arrestin is recruited to the GPCR, where it adapts its active conformation and sterically inhibits further interaction with the G protein. Once activated, β-arrestin takes up its second role as an adaptor for internalization of proteins, directing the GPCR-arrestin complex toward clathrin-coated pits for endocytosis [14, 15]. Recently, ARRBs have been recognized as signal transducers. ARRBs regulate three key signaling hubs (ERK-pathway, AKT-pathway, and NFκB-pathway), supporting cell growth and survival [16]. ARRB1 appears to be cardiotoxic in that genetic deletion of ARRB1 in the heart showed therapeutic effects in heart failure, whereas ARRB2 was found to be cardioprotective against cardiac inflammation and apoptosis [17]. Recent work has indicated that β-arrestin signaling is beneficial to the heart and may represent a novel therapeutic approach to prevent post-infarction pathological fibrosis and the transition to heart failure [18]. Intriguingly, ARRB1 is reported to act as a protective modulator during liver ischemia/reperfusion injury [16]. ELA is reported to protect against fibrosis, apoptosis and oxidative stress in the heart and kidney injury induced by ischemia–reperfusion[19] [12]. ELA, but not apelin, knockout in pregnant mice exhibited preeclampsia-like symptoms, including proteinuria and elevated blood pressure [10]. Our previous study found that ELA is exclusively expressed in human pluripotent stem cells and adult kidneys and can activate APJ signaling pathways, induce angiogenesis, and dilate mouse aortic blood vessels [20]. Our previous research also found that serum ELA levels gradually decreased with the deterioration of diabetic kidney disease [21]. But the underlying mechanisms are unknown. We speculate that the interaction of the APJ with ARRBs would mediate a part of ELA and apelin’s biological functions. However, little is known about the similarity and differences of ELA vs apelin in regulating the APJ-ARRB signaling pathway.

Methods of studying protein–protein interaction (PPI) include mainly immunocoprecipitation, fluorescence resonance energy transfer (FRET) and bioluminescence resonance energy transfer (BRET). Although widely employed, these methods have some drawbacks, such as difficulties in quantifying, low efficiency, multiple steps, and the requirement of special instruments. Recently, the NanoLuc® detection system (NanoBiT®) has been developed for protein–protein interaction analysis in live cells, which consists of two components of NanoLuc® luciferase, large N-terminal (LgBit) and small C-terminal (SmBit) region [22]. The LgBit and SmBit themselves have a low intrinsic association affinity, but when their tagged proteins interact in proximity, the two components will form an enzymatic entity to produce luciferase activity. The activity of NanoBiT® luciferase is linear, quantitative and stable for two hours in live cells, and the binding between SmBit and LgBit was reversible. Therefore, the technology can measure protein–protein interactions in real-time. Using the NanoBiT® technology, Storme J et al. found that the cytosolic portion of the adenosine A receptor (AAR), rather than the C-terminus alone, is required for the full activation of the receptor in respect of ARRBs recruitment [23]. Soave et al. have modified the human AAR on the N-terminus with the small high-affinity HiBiT tag for monitoring AAR [23]. In this study, we applied the NanoBiT® technique to compare the interaction of the APJ with ARRBs in response to ligands ELA and apelin.

## Materials and Methods

### Materials

The Nano-Glo Luciferase Assay System Kit with NanoLuc® substrate (furimazine) was purchased from Promega (Madison, WI, USA). The template plasmids of ARRB1/2 were purchased from Addgene (Cambridge, MA, USA). Apelin (apelin-13), ELA-14 and F13A were from American Peptide Company (Vista, CA, USA). Penicillin and streptomycin were from Ameresco (Solon, OH, USA).

### Plasmid Construction

**Fig. 1:**
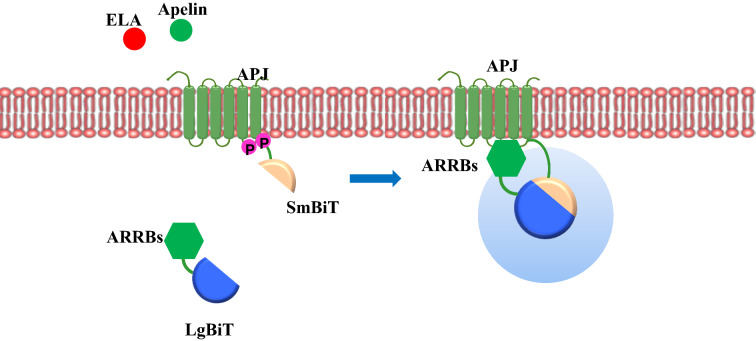
Schematic structures of NanoBiT®‐tagged constructs used in this study. APJ and ARRBs are fused to Lgbit or Smbit of NanoLuc® luciferase. When apelin or ELA binds to APJ, APJ recruits ARRBs in response to apelin or ELA, and the interaction yields a bright, luminescent signal in the presence of a substrate

The NanoBiT® system was implemented for the generation of fusion proteins consisting of the human APJ or ARRB1/2 fused via a peptide linker with the split subunits of NanoLuc® luciferase. In these fusion proteins, LgBit or SmBit was fused to the C-terminus of APJ and to the N-terminus of ARRB1/2 (Fig. 1). The human APJ cDNA fragment [20] was amplified by PCR with HindIII/EcoRI restriction sites and cloned into a HindIII/EcoRI-cut vector pBiT1.1-C (Promega, Madison, WI, USA) by Infusion cloning (Clontech, Mountain View, CA). Standard cloning protocol was used to fuse LgBit or SmBit to the N-terminus of ARRB1/2 by using XhoI/EcoRI cloning sites. All of the cDNA inserts were confirmed by restriction enzyme digestion and DNA sequencing. Vectors SmBit-PRKACA and LgBit-PRKAR2A (Promega) were used as a negative mock control. Primer sequences of APJ, ARRB1 and ARRB2 are listed in Table 1.

**Table 1:** The Primer sequence of target gene

Target gene	Primer sequence
APJ	F: TGAATCCAGAGGTTGATTGTCGACctaacctgcttctgctccagac
	R: GCAGCGACCCGCTTAAAAGCTTaccatggaggaaggtggtgatt
Arrb1	F: GGAGCGGAGGTGGAGGCTCGAGCggcgacaaagggacccgagtg
	R: ATCTGCTAGCTTAGACTGAATTCctatctgttgttgagctgtgg
Arrb2	F: GGAGCGGAGGTGGAGGCTCGAGCggggagaaacccgggaccagg
	R: ATCTGCTAGCTTAGACTGAATTCctagcagagttgatcatcata

The capitalized nucleotides are Infusion Cloning arms and the nucleotides in small letter are gene-specific sequences.

### Cell culture and Transfection

Human embryonic kidney (HEK) 293 T cells (ATCC, Manassas, VA) were cultured in complete Dulbecco’s modified Eagle medium (DMEM, Life Technologies, Grand Island, NY, USA), supplemented with 100 IU/ml of penicillin, 100 µg/ml of streptomycin and maintained at 37 °C, 5% CO_2_, under humidified atmosphere. Before transfection, 1 × 10^4^/well cells were seeded into a 96-well culture plate (Costar Cat.# 3917) in a total volume of 100 μl/well with complete DMEM. DNA plasmid was diluted with DMEM-free serum and antibiotics to 100 ng/well. Transfection of the indicated constructs was performed using CalFectin (Signagen, Gaithersburg, USA) with a 3:1 ratio of CalFectin:DNA. Healthy cells and gentle handling were required to prevent cells from detaching during transfection.

### NanoBiT® Based Binding Assay

Forty-eight hours post-transfection, the culture medium was replaced with serum-free DMEM (100 μl/well), and the cells were incubated for an additional 3.5 h. Diluted NanoLuc® substrate (a 20-fold dilution) for live cells (25 μl/well, Promega) was added to each well, and the luciferase activity was measured with 2030 Multilabel Reader (PerkinElmer VictorX3) before and after adding the substrate. Unless otherwise stated, ligand ELA or apelin was added at the concentration of 1 μM and luminescence was monitored for indicated times up to 60 min.

The interaction of the APJ with ARRBs induces the complementation of fused LgBiT and SmBiT, resulting in the reconstitution of NanoLuc® luciferase. The luminescence indicating the reconstituted NanoLuc® activity was measured in a total volume of 125 μL. For assay of the interaction between NanoBiT®-tagged APJ and ARRB1/2, 1 μM apelin or ELA was added to HEK293T cells transfected with tagged APJ and ARRBs. In the study of concentration-dependent interaction between APJ and ARRB induced by the ligands, ELA and apelin at six concentration levels (0.1 nM, 1n M, 10 nM, 100 nM, 1 μM, 10 μM) were used. For APJ inhibition study, F13A (1 μM) was added 5 min before apelin (1 μM) or ELA (1 μM) treatment.

### Data and Statistical Analysis

All data were expressed as mean ± standard error of the mean (SEM) of at least three independent experiments, each performed in triplicate. Statistical analyses were performed using GraphPad Prism 6 software (San Diego, CA, USA). EC_50_ values (drug concentration that produced 50% of its own maximal response) were determined, converted to their negative logarithms, and expressed as -log molar EC_50_. The data were analyzed using a Student’s *t*-test or a two-way analysis of variance with Tukey–Kramer multiple comparison tests for comparing multiple groups. A value of *P* < 0.05 was considered statistically significant.

## Results

### Determining the Optimal Pairing of Tagged APJ with ARRBs in Response to Ligand Treatment

**Fig. 2:**
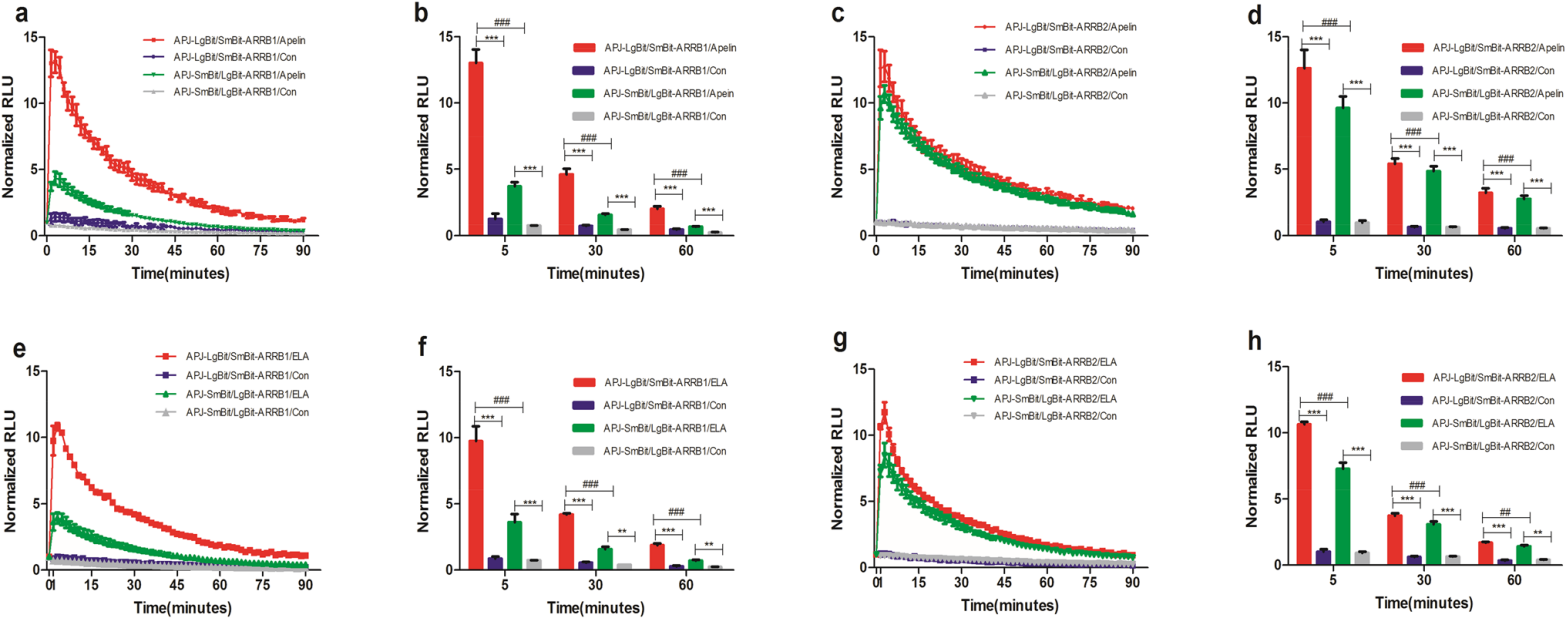
Pairing optimization of NanoBiT®-tagged APJ and ARRBs. **a**–**d**. NanoBiT®-tagged APJ- and ARRB1/2-expressing HEK293T cells were stimulated by apelin (1 μM) or PBS. **e**–**h**. NanoBiT®-tagged APJ- and ARRB1/2-expressing HEK293T cells were stimulated by ELA (1 μM) or PBS. Each value represents the mean ± SEM from 4 independent experiments. ^***^P < 0.001 and ^**^P < 0.01 vs. the corresponding control group; ^###^*P* < 0.001 and ^##^*P* < 0.01 vs. the corresponding APJ-LgBit/SmBit-ARRBs group. NS: no statistical significance

To analyze the effect of ELA and apelin on the interaction between APJ and ARRB1/2, we measured the interaction signal strength by varying combinations of tagged APJs (APJ-LgBit and APJ-SmBit) and tagged ARRB1/2 (LgBit-ARRB1, SmBit-ARRB1, LgBit-ARRB2, and SmBitARRB2) in HEK293T cells. As shown in Fig. 2a, b, compared with other groups, the combination of APJ-LgBit/SmBit-ARRB1 yielded the highest signal at the concentration of 1 μM for apelin. A similar result was obtained for APJ-LgBit/SmBit-ARRB2 (Fig. 2c, d). For ELA, the same combination produced the highest signal than other pairs (Fig. 2e–h). The results demonstrated that APJ-LgBit/SmBit-ARRB was the optimal combination to elicit a strong signal for interaction analyses. Therefore, the APJ-LgBit/SmBit-ARRB 1/2 was selected for subsequent experiments.

### The Effect of ELA or Apelin on the Interaction Between APJ andARRB1/2

**Fig. 3:**
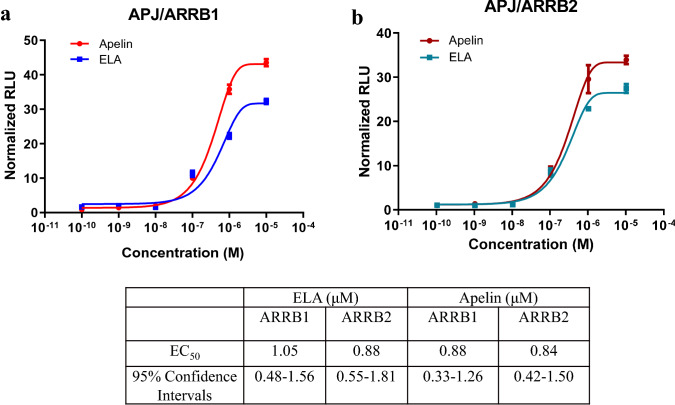
Concentration–response relationship of the interaction of APJ and ARRBs in response to ELA and apelin. **a**, indicated concentrations of apelin were added to living HEK293T cells transiently overexpressing APJ-LgBit/SmBit-ARRB1. **b**, indicated concentrations of ELA were added to live HEK293T cells transiently overexpressing APJ-LgBit/SmBit-ARRB2. EC_50_ was calculated from the concentration–response curve. Data are expressed as the mean ± SE (*n* = 6) and fitted to sigmoidal curves using GraphPad Prism 6 software

We next determined the concentration–response curves of the interaction by varying the ligand concentration from 0.1 nM to 10 μM. As shown in Fig. 3a, b, the NanoLuc® luciferase signal increased gradually with the ligand concentration. The EC_50_ values, indicating the concentration for 50% of the maximal effect, were calculated from these curves. The measured EC_50_ of ELA and apelin for the interaction of APJ/ARRB1/2 for ELA and apelin was similar, at the level of ~ 1 μM. However, apelin has a higher efficacy than ELA in activating the interaction of APJ with ARRB1/2 (Fig. 3b).

### Inhibition of APJ-ARRB Activation by F13A

**Fig. 4:**
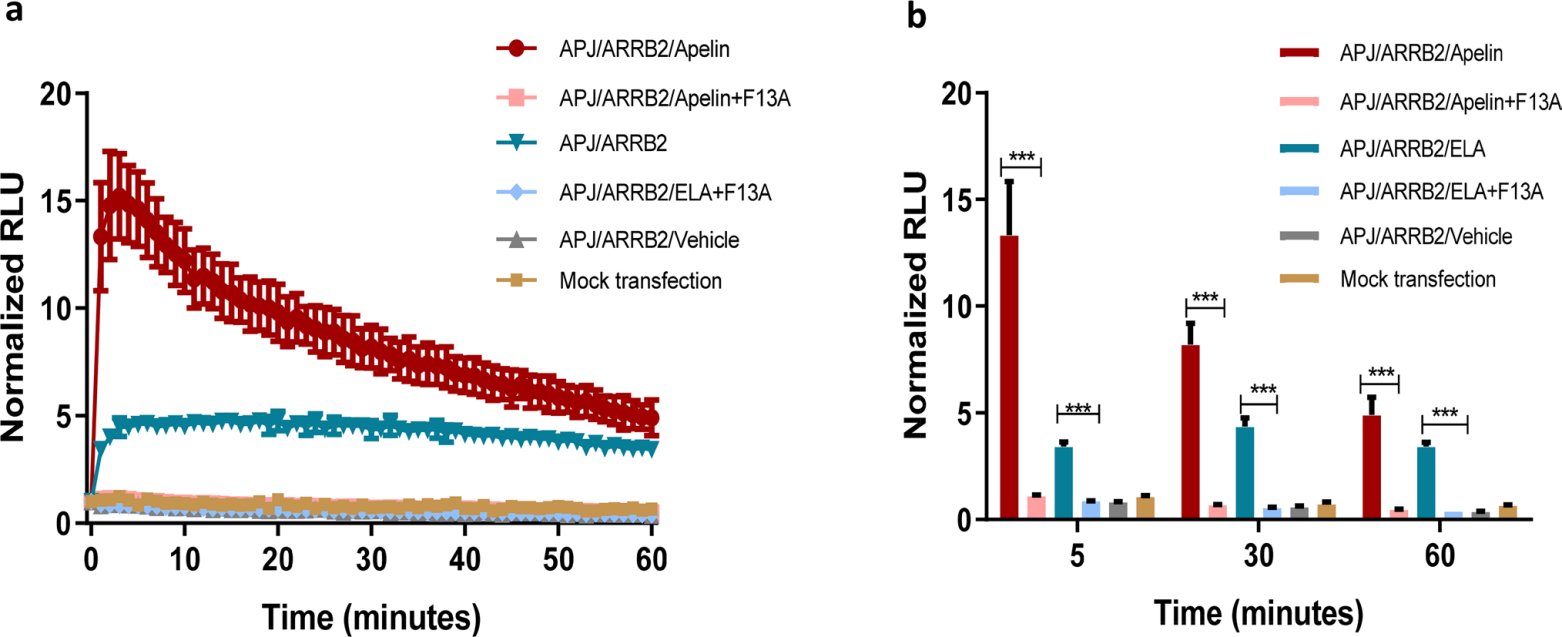
Inhibition of the APJ-ARRB interaction by F13A. **a**, **b,** Luciferase activities were measured in the cells pretreated with F13A (1uM) for 5 min, before the addition apelin or ELA. Each value represents the mean ± SEM from 6 independent experiments. ***P < 0.001. Control vectors SmBit-PRKACA and LgBit-PRKAR2A (Promega) were used as the negative control of no protein–protein interaction

To test whether an APJ antagonist suppressed ELA’s action on APJ, we pre-treated the transfected cells with F13A, an inhibitory apelin-13 analog, 5 min before apelin or ELA treatment. As is shown in Fig. 4a, b, the effect of apelin on the interaction between APJ and ARRB2 was nearly completely abolished, so was that of ELA. This study indicates that ELA and apelin may act through the shared site(s) on APJ.

## Discussion

In this study, we employed the NanoBiT® technology to compare the effects of ligands ELA and apelin on the interaction of APJ with ARRBs or the recruitment of ARRBs to APJ in live cells. As previous studies have reported on the interaction of the C-terminus of APJ with ARRBs, we tagged the NanoLuc’s subunits to the C-terminus of APJ. We found that the pairing of APJ-LgBit with SmBit-ARRB1/2 yielded a stronger signal than that of APJ-SmBit with LgBit-ARRB1/2. As the SmBiT is 1.3 kDa in size whereas the LgBiT is 18 kDa, this study indicates that the NanoBit® components may affect interactions of the parental proteins, and pilot experiments should be conducted for each protein pair to achieve optimal signal strength.

The activity of NanoBiT® reached the peak within 5 min after the addition of the ligand and then decreased with time, which is typical for A-type GPCRs [13]. We also found a concentration-dependent signal for both apelin and ELA with an EC_50_ of ~ 1 μm level. However, apelin was more efficacious than ELA in inducing the APJ-ARRBs interaction than ELA, which may suggest apelin induces more recruitment of ARRBs to APJ. Alternatively, the ARRB interaction with APJ induced by apelin may be more stable. The recruitment of ARRBs by GPCR mainly depends on two driving forces [24]: First, the ligand-activated receptor undergoes a conformational change, exposing the contact site of the receptor’s intracellular segment [25, 26]; Second, G protein-coupled receptor kinases, or second messenger kinases phosphorylate the C-terminal and/or intracellular loop of the receptor [27, 28]. Thus, apelin and ELA may differ in the ligand-induced conformational change. Further investigations are warranted about how the different APJ-ARRBs interactions elicited by ELA and apelin are related to their biological function.

Interestingly, Zhou S et al. reported that ELA exerts cardiovascular effects comparable to or greater than apelin [29]. One of the arrestin’s functions is known to desensitize GPCRs and to facilitate their degradation. Thus, the weaker efficacy of ELA than apelin in inducing the APJ-ARRB signaling may lead to less APJ desensitization, which may partially explain the stronger cardioprotective activity.

In the study, we found that pre-treatment with F13A, an APJ antagonist, nearly completely abolished apelin and ELA-mediated ARRB recruitment. Since F13A is an apelin analog with a phenylalanine-to-alanine substitution at the C-terminus and is structurally essentially the same as apelin-13, this result suggests that ELA may share the same or similar binding sites or pockets on the APJ as apelin-13.

In summary, we successfully employed the NanoBiT® technology to study the interaction of APJ with ARRBs in a live cell system. We found that ELA and apelin activate the APJ in a similar fashion but with differences with respect to ligand-mediated ligand-mediated ARRB recruitment and validated the inhibition of antagonist F13A to both apelin and ELA. This technology will be useful for identifying the protein residues implicated in the APJ-ARRB interactions.
